# Provisional Circulatory Support with Extracorporeal Membrane Oxygenation during Ventricular Tachycardia Ablation in Intermediate Risk Patients: A Case Series

**DOI:** 10.3390/jcm13154477

**Published:** 2024-07-31

**Authors:** Giuseppe Mascia, Luca Barca, Paolo Sartori, Daniele Bianco, Roberta Della Bona, Paolo Di Donna, Italo Porto

**Affiliations:** 1 Cardiovascular Disease Unit, IRCCS Ospedale Policlinico San Martino, 16132 Genova, Italy; dott.paolosartori@gmail.com (P.S.); daniele.bianco@hsanmartino.it (D.B.); roberta.dellabona@gmail.com (R.D.B.); didonnapaolo11@gmail.com (P.D.D.); italo.porto@gmail.com (I.P.); 2 Department of Internal Medicine, University of Genoa, 16145 Genoa, Italy; lucabarca1993@gmail.com

**Keywords:** cardiogenic shock, ventricular tachycardia, venoarterial extracorporeal membrane oxygenation, cardiac arrest

## Abstract

**Background:** Cardiogenic shock with acute hemodynamic decompensation may be one of the most serious risks in patients affected by ventricular tachycardia (VT). Its proper identification may have important implications in terms of pharmacological management, as might procedural planning in case of patients undergoing catheter ablation. **Methods:** We describe a case series of patients with provisional strategies for circulatory support in VT ablation, including the use of venoarterial extracorporeal membrane oxygenation (VA-ECMO) and vascular accesses in the electrophysiology lab but no initial ECMO activation due to an estimated intermediate pre-procedural risk from the case-series population. **Results:** In total, 10 patients (mean age 70 ± 11 years old, 9 males) with severe cardiomyopathy were admitted for incessant ventricular arrhythmia episodes, further diagnosis, and therapy planning; 1/10 patients (10%), documenting a PAINESD score of 14, underwent VA-ECMO cannulation due to electromechanical dissociation. All 10 patients were discharged alive. **Conclusions:** A pre-defined strategy before VT ablation is crucial. In our case series, the use of provisional circulatory support with VA-ECMO during incessant ablation of ventricular arrhythmia was a safe and winning alternative to upfront strategies.

## 1. Introduction

Despite significant improvements in ablation strategies for ventricular tachycardia (VT), the rate of ablation failure and recurrences remains high [1]. A variety of factors have been reported to increase the risk of unfavorable post-ablation outcome, potentially including an incomplete procedural success due to high number of clinical VTs or arrhythmic storm, a prolonged low-output state related to VT during procedure, as well as potential fluid overload due to irrigated catheter that may decompensate the patient status [1], increasing post-procedural mortality. Patients affected by VTs may also document several comorbidities, considering ischemic cardiomyopathy, idiopathic cardiomyopathy, or impaired left ventricular function [1,2] with increased risks and potentially life-threatening critical situations. In this scenario, cardiogenic shock with acute hemodynamic decompensation may be one of the most serious, and its proper identification may have important implications in terms of procedural planning since its treatment is extremely complicated and associated with a high mortality rate [3]. Upfront strategies with circulatory support using venoarterial extracorporeal membrane oxygenation (VA-ECMO) or, alternately, an Impella device have been used in high-risk patients undergoing VT ablation [4]. We describe a provisional strategy for circulatory support in VT ablation, which potentially includes the use of VA-ECMO vascular accesses in the electrophysiology lab but no initial ECMO cannulation.

## 2. Identifying Potential Risk of Acute Hemodynamic Decompensation during Catheter Ablation of Ventricular Tachycardia

Upfront identification of patients at the highest risk of hemodynamic decompensation has been determined using scores, representing tools in order to stratify the risk of peri-procedural acute decompensation in VT ablation, due to high burden comorbidities, complex underlying substrate and potential concomitant heart failure status [4]. The risk of acute hemodynamic decompensation has been recently predicted by clinical factors—in particular, pulmonary disease (P), age (A), ischemic cardiomyopathy (I), New York Heart Association class (N), ejection fraction (E), VT Storm (S), and diabetes (D)—defining the PAINESD score [4]. The risk of hemodynamic decompensation increased across tertiles of risk score, from 1% in the first tertile (≤8 points) to 6% in the second tertile (9–14 points), and finally to 24% for third tertile (≥15 points) [5,6]. However, while the PAINESD score may easily allow identification of patients at either very low or high risk, the intermediate-risk patients undergoing catheter ablation could be therefore somewhat extremely challenging, representing the aim of this case series. 

## 3. Case Series Results

In total, 10 patients (mean age 70 ± 11 years old, 9 males) with severe cardiomyopathy were retrospectively analyzed. Patients were admitted for incessant ventricular arrhythmia episodes, further diagnosis, therapy planning. Informed consent was obtained from all subjects. The study was conducted according to the guidelines of the Declaration of Helsinki and, as a retrospective, did not require ethical approval. A total of 4/10 (40%) patients documented a previous ischemic cardiomyopathy, while 6/10 (60%) documented a non-ischemic form. Table 1 summarizes characteristics. Coronary angiography was previously performed in the entire population, while only 2 non-ischemic patients underwent cardiac magnetic resonance due to clinical decision, documenting major criteria for arrhythmogenic cardiomyopathy and previous myocarditis, respectively (Table 1). All patients previously received implantable cardioverter–defibrillators (ICD) in primary prevention. Circulatory support was initially never considered in this population due to a pre-procedural intermediate-risk PAINESD score (documenting a score between 10 and 14, see Table 2). In this scenario, the Heart Team decided for a provisional VA-ECMO strategy as back-up circulatory support in hybrid electrophysiology lab, only achieving vascular accesses using high-support leads—no VA-ECMO cannulation was initially performed. The prepared vessels were the right common femoral artery and the left common femoral vein, and the size of the arterial and venous cannula was 21 French. Table 3 summarizes characteristics of procedural data.

Two patients underwent a redo procedure, and therefore, 12 VT procedures were analyzed (10 procedures were performed with an endocardial approach, 2 with a combination between endocardial and epicardial approaches). Mean procedural time was 240± 90 min, mean fluoroscopy time was 40 ± 19 min, and mean radiofrequency time was 35 ± 22 min. As peri-procedural complications, one patient (10%) had pericardial effusion that resolved spontaneously. In seven procedures (58.3%) a substrate-based VT ablation approach was performed, targeting abnormal electrograms and areas of slow and decremental conduction focusing on local abnormal ventricular activity (LAVA). In three procedures (25%) inducibility of VT was performed, while finally, two procedures underwent both approaches. At least one VT was acutely terminated in 100% of procedures with baseline inducible VT. Only one single patient (10%) with PAINESD intermediate-risk score (score: 14) underwent to VA-ECMO cannulation due to electromechanical dissociation during VT mapping. In this patient, an ECMO-supported ablation was the perfect bridge to acute VT ablation success. The hemodynamic support was not necessary in the remaining nine patients (90%) with intermediate-risk PAINESD scores. All patients were discharged alive, but one patient eventually died during follow-up because of mesenteric ischemia.

## 4. Case Description of the ECMO Supported Ablation

At admission, the patient was asymptomatic. Medical therapy: aspirin 100 mg, clopidogrel 75 mg, amiodarone 200 mg, bisoprolol 3.75 mg; furosemide 50 mg; canrenone 50 mg, ranolazine 375 mg, ezetimibe 10 mg, atorvastatin 40 mg, pantoprazole 40 mg. Laboratory chemistry revealed a significant rise in NT-proBNP (5374 ng/L), hemoglobin (Hb) 12 g/dL, creatinine 2.6 mg/dL and estimated glomerular filtration rate (eGFR) 22 mL/min/1.73 m^2^. Echocardiography showed a global left ventricular (LV) dysfunction with a reduced (27%) ejection fraction (EF), LV dilation, and moderate mitral regurgitation but no pulmonary hypertension. Device interrogation confirmed more than 100 VT episodes in the previous 6 months, with only 7 ICD shock interventions when VTs could degenerate into ventricular fibrillation (VF). Most VT episodes documented slow ventricular rate (125–135 beats per minute) and were hemodynamically tolerated. Cardiac angiography was carried out: severe coronary artery disease was confirmed, but no high-grade stenosis was documented. 

**Figure 1:**
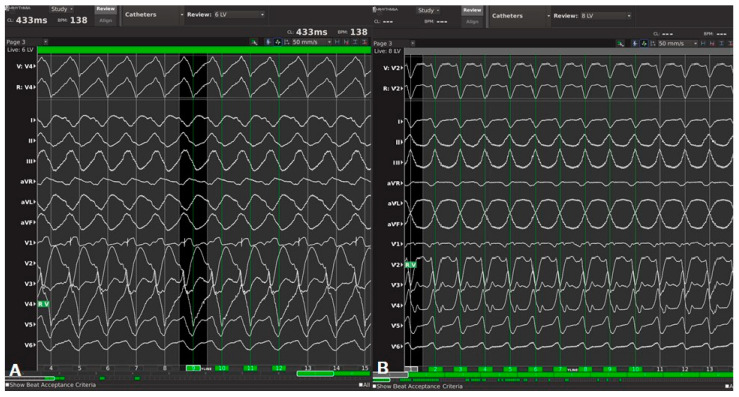
(**A**): 12-lead electrocardiogram (ECG) documenting an incessant VT with positive inferior leads and positive V1 (430 ms ventricular cycle length); (**B**): 12-lead electrocardiogram (ECG) documenting a second incessant VT morphology with positive inferior leads, negative V1, and V3 R/S transition (410 ms ventricular cycle length).

**Figure 2:**
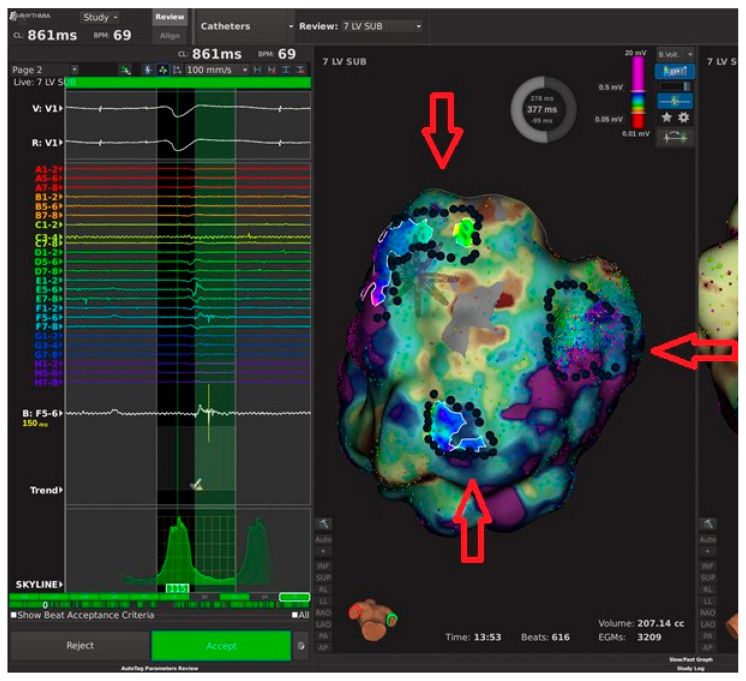
Voltage mapping revealing local abnormal ventricular activities (LAVA) and late potentials.

**Figure 3:**
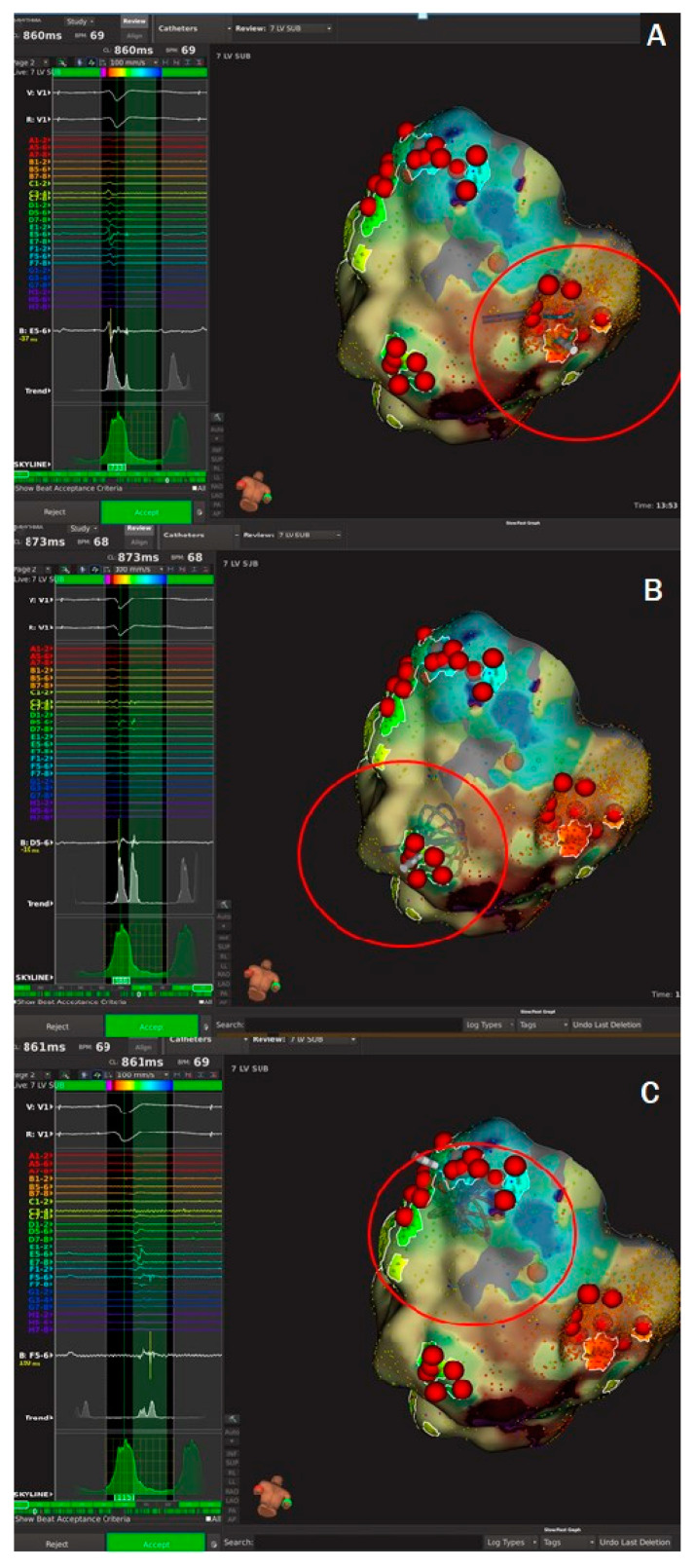
(**A**–**C**) clearly shows three different target-areas (red circles) of Local Abnormal Ventricular Activities (LAVA) and late potentials. The ablation was continued at each site (red tags) unless a local drop in impedance occurred (15–20 Ohm).

As discussed above, no circulatory support was initially considered due to pre-procedural risk of cardiogenic shock (PAINESD risk score: 14). The Heart Team decided on the provisional VA-ECMO strategy as backup circulatory support. The patient started the procedure in VT; in particular, atrio-ventricular dissociation in the coronary sinus activation was documented, and the 12-lead electrocardiogram (ECG) documented an incessant VT with positive inferior leads and positive V1 (430 ms ventricular cycle length, Figure 1A). Then, transeptal puncture was safely performed with anatomic approach using fluoroscopy, achieving left atrium access in a manner allowing safe passage of large-bore catheters, avoiding inadvertent trauma to adjacent structures, and providing a safe exit from the left atrium, avoiding tears in the interatrial septum (IAS) that could result in a larger-than-intended orifice with significant intracardiac shunting. No transesophageal echocardiography (TEE) and no intracardiac echocardiography (ICE) were performed. Then, a left-ventricular (LV) activation map during arrhythmia was created. Mapping points were collected from the Orion multipolar basket catheter (Rhythmia Mapping System, Boston Scientific, St Paul, MN, United States) with 64 electrodes of 0.4 mm^2^ area, and 2.5 mm inter-electrode spacing; more than 3200 intracardiac electrograms (EGMs) were collected. However, the initial arrhythmia rapidly changed into a second VT morphology with positive inferior leads but negative V1 and V3 R/S transition (410 ms ventricular cycle length, Figure 1B)—a second activation map during arrhythmia was created, while the patient became hemodynamically unstable (blood pressure 65/35 mmHg, oxygen saturation 80%). Electromechanical dissociation occurred since there was no effective cardiac output despite of continuing VT rhythm that suddenly could degenerate into VF (Supplementary Video S1). Then, cardiopulmonary resuscitation was initiated with cardiac massage, adrenaline, and finally external defibrillation (200 joule): hemodynamic response was optimal resulting in blood pressure 180/100 mmHg. After a careful 30 min evaluation with risk/benefit assessment, ECMO cannulation was initiated by an interventional cardiologist in a very short time (<15 min). This strategy, in association with the resulting stable and continuous high-density mapping, allowed conclusion of the procedure. The voltage map now revealed three different areas of local abnormal ventricular activities (LAVA) and late potentials (Figure 2). At this point, we used the Lumipoint™ algorithm [5], rapidly allowing identification of a specific EGM characteristic such as LAVA or late potentials and visualizing it within thousands of EGMs available. The activation map confirmed localized conduction within the three different areas. Radiofrequency ablation was then safely performed by means of a 4.5 mm tip catheter (Intella NAV MIFI OI; Boston Scientific, St Paul, MN, United States) in the three different target areas (Figure 3A–C: red circles). The ablation was continued from 30 to 60 s at each site (Figure 3A–C: red tags), unless a local drop in impedance occurred (15–20 Ohm) since was guided by DirectSense Technology, a feature based on local impedance, in order to guide the ablation phase according to the tissue response. Power was adjusted between 40 and 50 W (power control mode). At the end of the extended ablation protocol, a severe induction test was performed. The protocol consisted of a stimulation from the right ventricular apex up to three extra stimuli by decreasing the coupling interval until inducing sustained ventricular arrhythmias or reaching chamber refractoriness. No tachycardia was inducible. The patient demonstrated significant hemodynamic improvement immediately after procedure due to a stable and adequate perfusion pressure, blood flow, and oxygen delivery (blood pressure 120/80 mmHg, oxygen saturation 97%). The patient stayed on ECMO support for 150 min and was successfully weaned from ECMO before leaving the electrophysiology lab. Total percutaneous closure of the site of femoral arterial puncture with Perclose Proglide (PP) was performed. No complications related to ECMO cannulation were documented. The patient remained in the intensive care unit for 10 days under inotropic therapy and was finally discharged to rehabilitation after an additional 10 days. Transthoracic echocardiography showed good results and mild improvement of LVEF from 27% to 35%. No arrhythmic event was documented after inotropic therapy wash-out. After 30 days of follow-up, stable sinus rhythm was documented.

## 5. Discussion

The entire patient population from this case series had compromised LV function, remaining a challenge for VT ablation. In particular, tailoring of therapy to achieve hemodynamic goals is mandatory, focusing on cerebral oximetry, increases in pulmonary capillary wedge pressure, oliguria, increasing serum lactate, and sustained hypotension. A strict pre-procedural evaluation of patients is mandatory, determining heart failure performance as well as candidates who could benefit from mechanical hemodynamic support [7,8]. Many factors could impact hemodynamic status; some of these may be related to clinical presentation (as hypotension due to refractory arrhythmia or cardiac stunning due to repeated defibrillator shocks), while others could be modifiable (such as the use of vasopressors, inotropes, or general anesthesia during ablation) [9]. However, not only high-risk patients but also the intermediate-risk population with severe cardiomyopathy may be unlikely to tolerate the added risk of catheter ablation, and the option of circulatory support could help to benefit from catheter ablation. Prophylactic upfront placement of hemodynamic mechanical support in VT ablation has been clearly documented to prevent hemodynamic decompensation. Some authors proposed the use of Impella system therapy; these percutaneous and low-arterial-impact devices (14 French maximal diameter at the pump level) have been designed for temporary ventricular support in short-term use, reducing LV work and providing the circulatory support in order to allow early assessment of residual myocardial function [5,6,7,8,10,11]. Despite an axial continuous-flow pump delivering blood from LV to aorta up to 5 l/min, the Impella system during VT ablation may also show some disadvantages—in particular, anatomic hedge during LV mapping devices as well as the low support for right-sided heart failure, requiring preserved right ventricle function, should be considered. 

Other authors proposed prophylactic upfront placement of VA-ECMO as circulatory support in populations undergoing VT ablation with cardiogenic shock and refractory arrhythmia [12,13,14,15,16,17]. VA-ECMO was designed for long-term (up to several weeks) circulatory support with flow >5 L/min: this centrifugal continuous-flow pump with extracorporeal oxygenator providing CO > 4.5 L/min could also show disadvantages, considering the higher arterial impact (>21 French) and the higher cardiac afterload, potentially increasing LV volume and altering the quality of ablation mapping [18]. Most recent papers also propose prolonged hemodynamic support with ECMO in this setting [19,20,21]. In this scenario, costs associated with ECMO are an important factor in establishing cost effectiveness. ECMO therapy is an advanced and expensive technology, although reported costs differ considerably depending on ECMO indication and whether charges are measured. In particular, a large variation in hospital costs has been described, ranging from US $22,305 to US $334,608 [22]. Therefore, our strategy could potentially impact on cost effectiveness. 

In this case series, using the provisional VA-ECMO strategy as back-up circulatory support allowed vascular accesses using high-support leads, reaching a fast hemodynamic stability in less than 15 min when necessary (one single case). Our Heart Team’s pre-defined strategy was crucial in order to avoid both procedural failure and the patient’s death. In actuality, the value of VA-ECMO considering risks/benefits was not clear before the procedure (due to the intermediate PAINESD score), and to our knowledge, no study compared the different strategies in less-complicated settings. Few related VA-ECMO studies with small populations undergoing ablation with cardiogenic shock and refractory VT have been published [12,13,14,15,16,17]—in most, mortality remains high, although the majority of patients survived beyond 1 year [12,13]. In this scenario, modern ultra-high-resolution mapping systems allowed detection of the correct arrhythmic substrate [23,24], also considering awareness of radiation risks [25] in the era of near-zero X-Ray ablation [26]. In our case, the Lumipoint™ software, an automated algorithm, analyzed thousands of ECMs in a matter of seconds, eliminating the subjectivity of the human eye [23]. The three highlighted areas in Figure 3 demonstrate the presence of LAVA and late potentials, well represented in the white graph (trend tool), which shows two peaks—a first peak below the QRS and a second peak after the end of the QRS, revealing the target of the ablation (LAVA and late potentials). Accuracy was determined by the combination of a high-density mapping system (Rhythmia HDx) and the Orion multipolar basket catheter (64 electrodes of 0.4 mm^2^ area; 2.5 mm interelectrode spacing), allowing detection of even the smallest EGMs and rapidly elucidating the arrhythmic substrate in a complex and unstable scenario. All 10 patients were discharged alive.

## 6. Conclusions

The need of circulatory support, as well as its role in prophylactic strategy or in emergency scenarios, should be carefully evaluated from a Heart Team since most patients with cardiomyopathy may be unlikely to tolerate the added risk of catheter ablation. As the use of ECMO continues to evolve rapidly, this technology should not be used on unsalvageable patients. Future important directions are focused on the logistics of ECMO initiation, weaning, and ethical considerations. 

## Figures and Tables

**Table 1:** Patients’ characteristics.

ID	Date (d/m/y)	Age	Gender	EF %	ICD	Reason for ICD Implantation	Cardiovascular Drugs	Coronary Angiography
1	27 May 2020	71	M	25	Yes	Idiopathic dilated cardiomyopathy	Amiodarone, propranolol, furosemide, ARNI, MRA	Noncritical coronary stenoses on LAD
2	29 July 2020	80	M	20	Yes	Ischemic cardiomyopathy	Amiodarone, ASA, bisoprolol, statin, furosemide ARNI, MRA	Percutaneous coronary intervention of proximal and distal LAD with 2 DES
3	2 November 2020	80	M	27	Yes	Ischemic cardiomyopathy	Amiodarone, ASA, bisoprolol, statin	Percutaneous coronary intervention of RCA with 1 DES
4	14 April 2021	82	M	35	Yes	Ischemic cardiomyopathy	Amiodarone, ASA, bisoprolol, statin, NOAC	Critical coronary stenoses on proximal LAD and RCA undergoing CABG
5	26 May 2021	51	M	30	Yes	Arrhythmogenic cardiomyopathy	Bisoprolol, furosemide, ARNI, MRA	Negative
6	9 March 2022	51	M	20	Yes	Ischemic cardiomyopathy	Amiodarone, ASA, metoprolol, statin	Percutaneous coronary intervention of both proximal LAD and RCA with 2 DES
7	20 April 2022	82	M	45	Yes	Idiopathic dilated cardiomyopathy	Amiodarone, metoprolol	Noncritical coronary stenoses on RCA
8	11 May 2022	80	F	50	Yes	Previous Myocarditis	Amiodarone, bisoprolol	Negative
9	26 October 2022	65	M	35	Yes	Idiopathic dilated cardiomyopathy	Amiodarone, metoprolol, mexiletine, NOAC furosemide, ARNI, MRA, 5GLT2i	Negative
10	14 June 2023	76	M	30	Yes	Idiopathic dilated cardiomyopathy	Amiodarone, metoprolol, furosemide, ARNI, MRA, 5GLT2i	Noncritical coronary stenoses on LAD and RCA

EF: ejection fraction; ICD: implantable cardioverter defibrillator; ARNI: angiotensin receptor neprilysin inhibitor; MRA: mineralcorticoid receptor antagonist; 5GLT2i: sodium glucose co-transporter 2 inhibitors; NOAC: non vitamin K antagonist anticoagulant; LAD: left anterior descending artery; RCA: right coronary artery; DES: drug eluting stent; CABG: coronary artery bypass graft.

**Table 2:** PAINESD score data.

ID	PAINESD Score	Variables in PAINESD Score
1	12	(A) Age > 60: 3 points (N) NHYA class III: 3 points (E) EF<25%: 3 points (D) Diabetes:3 points
2	12	(A)Age > 60: 3 points (I) Ischemic disease: 6 points (E) EF<25%: 3 points
3	14	(A)Age > 60: 3 points (I) Ischemic disease: 6 points (S) Storm VT: 5 points
4	14	(P) Pulmonary disease: 5 points (A) Age > 60: 3 points (I) Ischemic disease: 6 points
5	10	(P) Pulmonary disease: 5 points (S) Storm VT: 5 points
6	14	(P) Pulmonary disease: 5 points (I) Ischemic disease: 6 points (E) EF<25%: 3 points
7	11	(A) Age > 60: 3 points (N) NHYA class III: 3 points (S) Storm VT: 5 points
8	11	(A) Age > 60: 3 points (S) Storm VT: 5 points (D) Diabetes: 3 points
9	11	(A) Age > 60: 3 points (N) NHYA class III: 3 points (S) Storm VT: 5 points
10	13	(P) Pulmonary disease: 5 points (A) Age > 60: 3 points (S) Storm VT: 5 points

**Table 3:** Procedural data.

ID	Date	Epicardial Ablation	VT Cycle Length (ms)	Total Procedure Duration (min)	Type of Ablation	ECMO	VTs Induced	Periprocedural Complications
1	27 May 2020	No	360	280	arrhythmia + substrate	No	yes	No
2	29 July 2020	No	375	250	Substrate	No	yes	No
3	2 November 2020	No	410	430	Substrate	Yes	yes	electromechanical dissociation
4	14 April 2021	No	290	280	Substrate	No	yes	No
5	26 May 2021	No	330	200	Substrate	No	yes	no
5 (redo)	11 August 2021	No	190	255	Substrate	No	yes	no
6	9 March 2022	No	340	220	arrhythmia + substrate	No	yes	no
7	20 April 2022	Yes	370	330	Substrate	No	yes	no
8	11 May 2022	No	350	180	arrhythmia	No	not performed	pericardial effusion
9	26 October 2022	No	430	240	arrhythmia	No	no	no
9 (redo)	7 November 2022	Yes	330	140	arrhythmia	No	yes	no
10	14 June 2023	No	270	260	Substrate	No	yes	no

## Data Availability

The data presented in the study are available on request from the corresponding author.

## Supplementary Materials

The following supporting information can be downloaded at: https://www.mdpi.com/article/10.3390/jcm13154477/s1**.**
